# Trends in the representation of women in the American Society of Pediatric Nephrology program at the Pediatric Academic Society annual meetings 2012–2021

**DOI:** 10.3389/fped.2023.1185329

**Published:** 2023-04-27

**Authors:** Bahar Barani-Najafabadi, David T. Selewski, Danielle E. Soranno, Darcy K. Weidemann

**Affiliations:** ^1^ Children's Mercy Kansas City, Department of Graduate Medical Education, Kansas City, MO, United States; ^2^ Department of Pediatrics, Medical University of South Carolina, Charleston, SC, United States; ^3^ Department of Pediatrics, Indiana University School of Medicine, Indianapolis, IN, United States; ^4^ Children's Mercy Kansas City, Department of Pediatrics, Division of Nephrology, Kansas City, MO, United States; ^5^ Department of Pediatrics, University of Missouri—Kansas City School of Medicine, Kansas City, MO, United States

**Keywords:** diversity, pediatric nephrology, presentation of women, equity, trends, pediatrics, representation

## Abstract

**Introduction:**

Women are under-represented in virtually all fields of academic medicine. Even in pediatrics, a field that historically attracts a workforce with a majority of women physicians, substantial gender disparities persist in leadership positions. However, previous studies of gender representation in various academic settings are limited to small studies or aggregate pediatric subspecialties, thereby omitting important granularity within each subspecialty. No prior studies have investigated potential gender disparities in pediatric nephrology. The aim of this study is to determine the representation of women physicians in leadership and speaking roles in the annual American Society of Pediatric Nephrology (ASPN) meeting.

**Methods:**

Data were analyzed from the 2012–2022 ASPN annual scientific meetings at the Pediatric Academic Society (PAS). Data were abstracted regarding gender and roles: speaker, chair/moderator, and lifetime achievement awardee. We performed a time series analysis using linear regression, with the year as the independent variable and the proportion of women as the dependent variable.

**Results:**

Overall, there were statistically significant increases in the proportion of women speakers per year and percentage of women chairs or moderators. There were no specific trends noted for lifetime achievement awards and no statistically significant changes in the number of lifetime achievement awards.

**Discussion:**

We found proportionate representations of gender representation with regards to speakers and chairs or moderators, although our data was limited by comparison to the American Board of Pediatrics (ABP) workforce cumulative “ever certified” data. The ABP data include a disproportionate representation of faculty who are men from earlier certification periods who may no longer be actively practicing pediatric nephrology.

## Introduction

Disparities in physician women leadership roles persist despite multiple publications addressing the under-representation of women in academic medicine (1, 2). Although gender parity has existed within medical school graduates for over 20 years, the 2021 Association of American Medical Colleges reports that women constitute 43% of full-time academic faculty members, only 28% of full professors, 22% of department chairs, and 18% of deans (3). The so-called “leaky pathway” can be attributed to a myriad of factors including lack of mentorship and sponsorship, inequitable distribution of time-consuming administrative tasks, institutional barriers, implicit bias, and gender discriminatory policies (4, 5). Although the field of pediatrics represents a unique situation in which women residency graduates now comprise nearly 75% of the graduating cohort, with some notable successes for women pediatrician leaders, many challenges remain (6).

Speaking opportunities at national scientific meetings are important academic currency required for career advancement and academic promotion. Previous studies of national conference invited speaker lists demonstrate modest improvements in gender diversity over time for all specialties in aggregate (7, 8), yet lagging when compared to overall workforce data. A study performed by adult nephrology members of the American Society of Nephrology annual Kidney Week national meeting found an uptrend over the past decade of women moderators and speakers but persistently low number of women early program chairs and lifetime achievement award winners (9). Although multiple subspecialty societies are starting to examine and publish their own internal national meetings to determine gender parity, no previous investigations exist within pediatric subspecialties either as a whole, or within pediatric nephrology.

This study aims to evaluate trends in women faculty representation at American Society of Pediatric Nephrology (ASPN) annual scientific meeting. Data on this topic is crucial to determine whether speaking roles are equitably distributed between men and women, which is an important contributor to equalizing professional advancement opportunities in the field of pediatric nephrology.

## Methods

ASPN conference agenda lists from 2012 to 2022 were obtained for the purpose of this analysis. Specific data abstracted included conference attendees' gender and roles at the conference. Gender was determined based on the individual's name. When the gender was indeterminant based on name, two separate internet-based searches for the individual's image and gender-identifying data were performed. Roles analyzed included speakers, chair/moderator, and lifetime achievement awards.

The proportions were compared to data from the American Board of Pediatrics Subspecialty Certification Database “Ever Certified” which includes all ABP Diplomates since pediatric nephrology became a specialty in 1974. The ASPN has not collected gender data of its membership since 2017, although a sensitivity analysis was performed comparing to the ASPN membership directory for years 2017–2021. We performed a time series analysis using linear regression, with the year as the independent variable and the proportion of women as the dependent variable. Representation ratios were calculated for speaker, chair/moderator, and awardee roles. All data analysis were performed with Stata (StataCorp release 17.0, College Station, TX). *P* < 0.05 was considered statistically significant.

## Results

**Figure 1:**
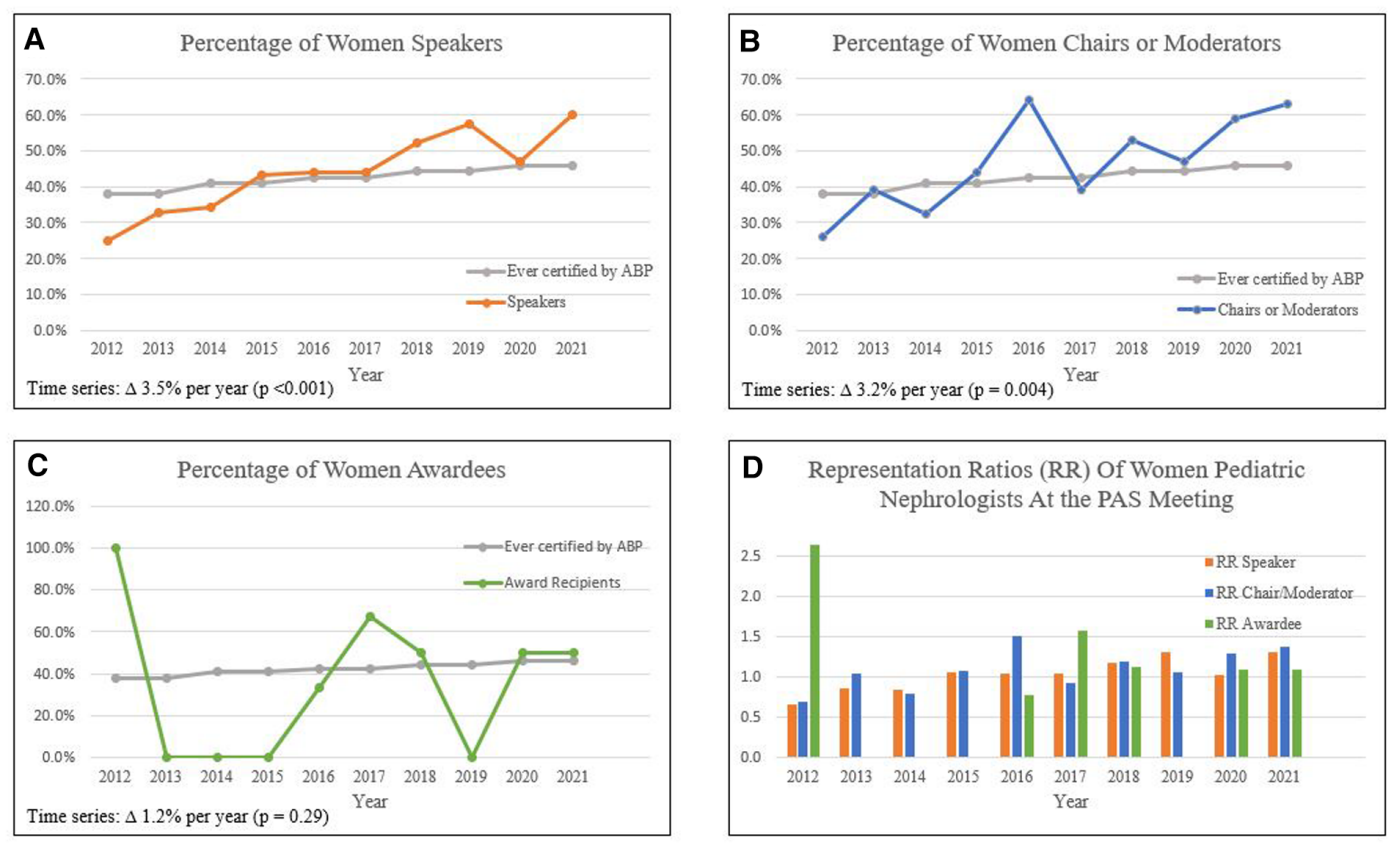
Proportion of women speakers at ASPN PAS meetings, 2012-2021. (**A**) Percentage of women speakers over time showed significant increase in 3.5% per year (*p* < 0.001) using time series linear regression analysis. (**B**) Percentage of women chairs or moderators increased 3.2% per year (*p* = 0.004). (**C**) Percentage of women awardees showed no statistically different trends over time (*p* = 0.29). (**D**) Representation ratios over time for speaker, chair/moderator, and awardee roles.

A total of 560 speaker positions, 279 chair or moderator positions, and 23 lifetime achievement awards were available during the time period of study. The proportion of women speakers increased over 10 years, with a total representation of 46.9% which was proportionate to the overall workforce of 45.9% women ever certified by the ABP in pediatric nephrology. Figure 1 depicts the time series analysis for women speakers (Panel A), chairs or moderators (Panel B), and lifetime achievement awardees (Panel C). Time series analysis showed that the proportion of women speakers increased significantly by 3.5% per year (*p* < 0.001, 95% CI 1.9 to 5.1%). The percentage of women chairs or moderators also increased over time, from 26.1% in 2012 up to 63% in 2021. Time series analysis demonstrated that the proportion of women chairs or moderators increased by 3.2% over time (*p* = 0.004, 95% CI 1.0% to 5.3%). A total of 8 women have received lifetime achievement awards over the past 10 years, which is 34.8% of the total awards (*n* = 23) given, not statistically significant based on Chi-square analysis (difference 11.1%, 95% CI −9.4% to 27.3%, *p* = 0.29). No trend was detected for lifetime achievement awards with linear regression (1.2%, *p* = 0.741, 95% CI -.065%–9%). Panel D depicts representation ratios for all three variables, with the numerator encompassing percentage of women invitees and the denominator representing percentage of women ever certified by the ABP. A representation ratio approximately equal to 1.0 indicates gender parity, with a representation ratio > 1 indicating overrepresentation of women speakers compared to men. ASPN membership demographics were only available for a subset between 2017 and 2021 although were generally congruent with the ABP data, with slightly lower percentages of women members (Table 1). This resulted in slightly higher representation ratios for all years studied.

**Table 1:** Comparison of representation ratios (RR) using the ASPN membership database proportion of women pediatric nephrologists, compared to proportion of women pediatric nephrologists in the ABP ever certified database.

	ASPN membership database				ABP “ever certified” database			
Year	% women	RR speaker	RR chair/moderator	RR awardees	% women	RR speaker	RR chair/moderator	RR awardees
2017	30%	1.5	1.3	2.2	42.5%	1.0	0.9	1.6
2018	37%	1.4	1.4	1.4	44.3%	1.2	1.2	1.1
2019	38%	1.5	1.2	0	44.3%	1.3	1.1	0.0
2020	39%	1.2	1.5	1.3	45.9%	1.0	1.3	1.1
2021	46%	1.3	1.4	1.1	45.9%	1.3	1.4	1.1

## Discussion

This study aimed to describe the representation of women speakers at a major pediatric nephrology academic conference. Overall, the study showed statistically significant increases in the proportion of women invited as speakers and women as chairs or moderators from 2012 to 2022, and achievement of gender parity for all roles by 2018. Although a trend was noted towards an increase in the number of lifetime achievement awards given to women, this was not statistically significant. This is the first study to assess for gender disparities in national speaking positions at a national pediatric nephrology conference.

A growing body of literature demonstrates some improvements in gender equity in national speaking roles for academic conferences for critical care (10), anesthesiology (11), and orthopedic surgery (12). Prior cross-sectional studies examining gender diversity at major medical specialty conferences have noted significant underrepresentation of women, although the trend no longer reached statistical significance by 2017 (6). The most comprehensive study thus far in the literature by Ruzycki et al. assessed gender representation in > 700 conferences in North America over a decade and found that the proportion of women speakers overall increased from 25% to 34% (7). A higher proportion of women on a planning committee was associated with a greater number of women speakers (13), indicating that a possible solution to gender disparities may derive from greater gender balance within planning committees. Use of validated tools (14) for monitoring gender representation at academic conferences can also provide important accountability and transparency to the planning organizations.

While our study does show increases in representation over time, the study may be limited by an unknown “true” denominator of practicing women pediatric nephrologists. This is because the data provided by ABP is cumulative for “ever certified” since pediatric nephrology became a specialty in 1974 and thus may include a disproportionate number of faculty who are men who may be retired. However, comparison to 5 years' worth of demographic data from the ASPN membership directory indicated that the proportion of women representation within the ASPN was actually less than the gender percentages provided by the ABP between −12.5% and +0.1%, making our representation ratios likely valid inferences. Secondly, the means to classify gender for this study was limited given we did not have access to self-identified gender identity. Thus, assumptions and inferences were made about an individual's gender which may have introduced bias into the study.

Future studies would benefit from obtaining self-reported gender identity information, in addition to race and ethnicity which are also important aspects of achieving diversity and equity in academic medicine and were not addressed within this study. However, this study represents an important first step in understanding the gender diversity within national academic meetings within pediatric nephrology. Acknowledging existing disparities is an important first step in addressing barriers to academic leadership roles for women in pediatric nephrology. Future dedicated efforts in rebalancing gender gaps in academic professional society meetings are the collective responsibility of conference organizers, speakers, and attendees. Organizers should be mindful and transparent of the panels and speakers when selecting speakers with relevant expertise, offer equal speaking fees for men and women, engage early with speaker invitations to allow adequate time to provide for leave or childcare arrangements, and consider offering coaching for more inexperienced speakers. Attendees should be cognizant if unbalanced speaker lineups occur and highlight perceived disparity to the conference organizers and wider community. Women speakers should ensure their voice is heard on mixed-gender panels, and consider coaching junior colleagues or mentees to promote future speaking opportunities.

## Data Availability

The raw data supporting the conclusions of this article will be made available by the authors, without undue reservation.
